# Typhoid among the Vaccinated

**Published:** 1916-03

**Authors:** 


**Typhoid Among the Vaccinated.** Andre Tournade, Presse Med., Nov. 25, 1915, reports 142 cases among soldiers. The course was usually mild, the mortality 7% as compared with 14% among those not vaccinated. There were no deaths among those who had received 4 inoculations. (Note: Nevertheless, this report and the following show that anti-typhoid vacination is by no means the ''humanly speaking'' perfect protection which has been claimed by enthusiasts.)

L. Rimbaud, Presse Med., Nov. 11, 1915, made 656 blood cultures between Dec. 15, 1914 and April 30, 1915. 339 (51.6%) were positive. Of the 339 positive cases, 164 were shown to be true typhoid, 121 paratyphoid A, 54 paratyphoid B. Among 434 subjects who had received 1-4 antityphoid inoculations, 1 month to over 2 years previously, 203 blood cultures (46.7%) were positive, true typhoid being found in 51, paratyphoid A in 106, paratyphoid B in 46. Of the 51 cases of typhoid, 4 had received one injection; 20 2; 17, 3; 10, 4. He therefore urges 4 injections as a routine.

Marcel Labbe, Le Progres Med., Dec. 1915, reports 137 cases of typhoid and paratyphoid among those vaccinated, and checked by blood cultures, sero-diagnosis and cultures from faeces and bile. 1-injection cases showed 50% each of typhoid and paratyphoid B; 2-injection cases, 9.5% typhoid, 90.5% paratyphoid B; 3-injection cases, 8.5% typhoid, 91.5% paratyphoid A. Among the unvaccinated, 87.8% of the fevers were typhoid, 10.9% paratyphoid B, 1.1% paratyphoid A. The mortality of typhoid was: among non-vaccinated 21.4%, among 1-injection vaccinated 12.5%, among those injected twice or more, 2.4%.

Leon Bernard, Le Progres Med., Nov. 1915, states the mortality of typhoid among the non-vaccinated to be 17.2%, among the vaccinated 5.6%. In fact the latter mortality is entirely due to paratyphoid. Hence he urges vaccination with triple vaccine.

Am. Coyon and Lucien Rivet (ibid) confirm the general conception of the relative benignity of paratyphoid infections, though recognizing their menace against military efficiency. As treatment, they recommend ice to the abdomen, adrenalin 30 drops daily, sulphur 1.20 t.i.d., daily intestinal lavage with 500 C.C. of water (boiled) plus 20 C.C. Labarracque solution. In 9 cases, they used colloid gold intravenously, with good results.

Petges, Dumoyra and Peyri, Jour. de Med. de Bordeaux, Nov. 1915, report 330 cases of typhoid, well marked clinically but apparently not definitely differentiated from paratyphoid. 278 cases were studied in detail, the mortality being 14.39%. 38 cases occurred among the vaccinated, 9 having had 1 injection; 17, 2; 7, 3; 5, 4 injections. 4 deaths occurred, 2 having had 2 injections, 1, 4 injections. The mortality was over 15% in the non-vaccinated, a trifle less than 8% in the vaccinated.

